# Phytochemical Investigation on *Euphorbia macrostegia *(Persian wood spurge)

**Published:** 2015

**Authors:** Somayeh Zare, Mehrorang Ghaedi, Ramin miri, Sven Heiling, Mojtaba Asadollahi, Ian T. Baldwin, Amir Reza Jassbi

**Affiliations:** a *Medicinal and Natural Products Chemistry Research Center, Shiraz University of Medical Sciences, Shiraz, Iran.*; b *Department of Phytochemistry, Yasouj University, Yasouj 75914-353, Iran. *; c *Department of Molecular Ecology, Max Planck Institute for Chemical Ecology, Hans-Knöll-Strasse 8, D-07745 Jena, Germany.*

**Keywords:** Euphorbiaceae, *Euphorbia macrostegia*, Cycloartane triterpenoids, Diglycerides of fatty acids

## Abstract

*Euphorbia macrostegia* or Persian wood spurge is one of the seventeen endemic plants of this genus in Iran. Three triterpenoids, 24-methylenecycloartan-3β-ol (1), butyrospermol (2) and cycloartenol (3) and three diglycerides, 1,2-di-*O*-α-linolenoyl-*sn*-glycerol (4), 1-*O*-linoleoyl-3-*O*-palmitoyl-*sn*-glycerol (5) and 1-*O*-α-linolenoyl-2-*O*-palmitoyl-*sn*-glycerol (6) were isolated from the hexane soluble part of methanol-dichloromethane extracts of the aerial parts of *Euphorbia macrostegia *Boiss. The structures of all compounds were elucidated using different spectroscopy methods including, ^1^H NMR, ^13^C NMR, HSQC, HMBC, EI-MS and IR. The triterpenes and the unsaturated fatty acids moieties of the diglycerides isolated from the plant were reported previously to have analgesic, anticancer, bactericidal and antifungal activity. Here, we show that *E. macrostegia *is a new source for the above mentioned biologically active compounds.

## Introduction

*Euphorbia macrostegia* (Persian wood spurge) is one of the seventeen endemic plants of this genus in Iran (1). It grows wild in different parts of Iran including Fars, Kohgiluyeh and Boyer-Ahmad Provinces (2). The plants of the genus *Euphorbia* are known to have cycloartane triterpenoids, long chain hydrocarbons and fatty acids as their major non-polar constituents (3).

The cycloartane triterpenoids are one of the main groups of the natural products with interesting biological activities including cytotoxic (4), anti-inflammatory (5), and antimicrobial properties (6,7) and are important in the chemical classification of the genus *Euphorbia *(8). Antitumor, skin-irritant macrocyclic- and polycyclic diterpenoids and triterpenoids are common secondary metabolites of different *Euphorbia *species; while fatty acid glycerides are less reported from the genus *Euphorbia*. In this paper, we report the isolation of three triterpenoids, 24-methylenecycloartan-3β-ol (1), butyrospermol (2) and cycloartenol (3) and three diglycerides, 1,2-di-*O*-α-linolenoyl-*sn*-glycerol (4), 1-*O*-linoleoyl-3-*O*-palmitoyl-*sn*-glycerol (5) and 1-*O*-α-linolenoyl-2-*O*-palmitoyl-*sn*-glycerol (6) from methanol and dichloromethane extract of the aerial parts of *Euphorbia macrostegia* Boiss (3,9). Recently, four cycloartane triterpenoids were isolated from dichloromethane extract of *E. macrostegia* (10) among which 24-methylenecycloartan-3β-ol was the only common compound with our report*.* The cytotoxic activities of the isolated compounds on two human cancer cell lines were determined, but the less polar triterpenoids, 24-methylenecycloartan-3β-ol did not show high anticancer activity against the tested cell lines (10).

## Experimental

*General*

The puriﬁed compounds were subjected to NMR measurements on Bruker Avance DRX500 Spectrometer (500 MHz for ^1^H and 125 MHz for ^13^C).^ 1^H- and ^13^C-NMR were measured in CDCl_3_ with TMS as internal standard. Mass spectra (EI-MS) were recorded on an Agilent 5975C inert GC/MSD instrument. IR spectra were recorded on a Perkin Elmer Spectrum One FT-IR spectrometer in CHCl_3_. The chromatography separations were performed using gravity columns chromatography with silica gel 60 (70-230 mesh, 0.063-0.200 mm particle size) and using flash column chromatography (FCC) with silica gel 60 (230-400 mesh, 0.040-0.063-mm particle size) and thin layer chromatography (TLC) using silica gel 60 F_254_ pre-coated plates (0.25 mm). The above adsorbents were purchased from Merck Chemical Company, Darmstadt, Germany.

*Plant material*

The aerial parts of *E. macrostegia* Bioss. in the flowering stage were collected from Chelegah (N 30º 17', E 51° 56'; at 2370 m altitude ) near Sepidan (Ardakan-e-Fars) Fars, Iran, in July 2012 and the plant was identified by Mojtaba Asadollahi in Medicinal and Natural Product Chemistry Research Center (MNCRC), Shiraz University of Medical Sciences. A voucher specimen (PC-91-4-11) has been deposited at the herbarium of (MNCRC).

*Extraction and fractionation*

The air-dried powdered plant (310 g) was extracted successively in DCM (3 L) and MeOH (3 L) by maceration for eight days at room temperature. The filtered extracts were concentrated under reduced pressure by rotary evaporator to afford residues of MeOH (9 g) and DCM (6 g) extracts. The crude extracts were mixed together and subjected to liquid-liquid extraction (LLE). The LLE has afforded four different phases, from non-polar to polar: *n*-hexane (5.3 g), DCM (0.9 g), 1-butanol and water. On the basis of TLC examination, we choose the hexane fraction for further phytochemical investigation.

*Isolation of compounds from the hexane fraction*

The hexane extract was subjected to column chromatography (50 × 5 cm) over silica gel (150 g, 70-230 mesh). The elution of the column was performed using *n*-hexane with 10% gradient of DCM up to 100%, and then followed by increasing the polarity of the mobile phase with MeOH to afford 48 fractions. Similar fractions were combined based on their similarity in composition, deduced from TLC analyses. Fractions 17-19 (570 mg) were combined and subjected to 5% AgNO_3_-silica gel (60 g) impregnated FCC (30 ×3 cm) (11). The column was eluted with DCM with increasing the polarity to ethyl acetate (EtOAc) that yielded compounds 1 (15 mg) and 2 (45 mg) as a mixture (their ratios were calculated on the basis of their proton's signal integration in the ^1^H NMR spectrum) and 3 (20 mg). Fractions 24-27 (170 mg) were pooled and were separated on a FCC (17 g, 20 × 2 cm) using DCM with a gradual increasing of the polarity with EtOAc as the mobile phase. Compound 4 (7.2 mg) was purified from the above column. Fractions 28 to 32 (1.4 g) were mixed and loaded on a flash silica gel column (100 g, 50 × 4 cm) and eluted with DCM with a gradient of the polarity to acetone. It afforded 40 fractions. Fraction mixture 17-28 (70 mg) was further purified by repeated FCC to yield compound 5 (9.1 mg) and 6 (16.2 mg).

*Spectral data*

The structures of all compounds isolated from *E. macrostegia* were elucidated using spectroscopy methods including ^1^H NMR, ^13^C NMR, EIMS, IR and by comparison of their spectra with those published in the literature for the authentic samples (5,8,12-16).

*24-methylenecycloartan-3β-ol (1)*: White amorphous powder (15 mg) R_f_ 0.41 (5% AgNO_3_-silica gel TLC, DCM: EtOAc 95:5). IR ν_max_ cm^-1^: 3054(=C-H) (cyclopropane ring), 2987(C-H), 1712, 1602, 1421, 1265 (C-O), 739, 705; EIMS (*rel. int.*%): *m/z* 440[M]^+^C_31_H_52_O (8), 425 [M-15]^+^ (12), 407 [425-18]^+^(15), 315 [M-side chain]^+^, 297 [315-18]^+ ^(7), 300 (12), 175 (25). ^1^H NMR (500 MHz, CDCl_3_):δ_H_ 4.72 ppm (1H, brs, H-31), 4.66 (1H, brs, H-31), 3.28 (1H, m, H-3), 1.03 (3H, d, *J* = 6.9Hz), 1.02 (3H, d, *J* = 6.8, H-26), 0.97 (6H, s, H-18, H-30), 0.90 (3H, brs, H-21), 0.90 (3H, s, H-29), 0.81 (3H, s, H-28), 0.55 (1H, d, *J* = 4.0 Hz, H-19b), 0.33 (1H, d, *J* = 4 Hz, H-19a).^13^C NMR (125 MHz, CDCl_3_): δ_C_ 31.9 (C-1), 30.4 (C-2), 78.8 (C-3), 40.5 (C-4), 47.1 (C-5), 21.1 (C-6), 28.2 (C-7), 48.0 (C-8), 20.0 (C-9), 25.2 (C-10), 26.0 (C-11), 32.9 (C-12), 45.3 (C-13), 48.8 (C-14), 34.9 (C-15), 26.5 (C-16), 52.3 (C-17), 18.0 (C-18), 29.9 (C-19), 36.1 (C-20), 18.3 (C-21), 35.6 (C-22), 31.3 (C-23), 156.9 (C-24), 33.9 (C-25), 22.0 (C-26), 19.3 (C-27), 18.3 (C-28), 14.0 (C-29), 25.4 (C-30), 105.8 (C-31) (5,8,12).

*Butyrospermol (2)*: White amorphous powder (45 mg) R_f_ 0.41. (5% AgNO_3_-silica gel TLC, DCM: EtOAc 95:5). IRν_max_ cm^-1^: 3054(=C-H), 2987 (C-H), 1712, 1602, 1421, 1265 (C-O), 739, 705; EIMS (*rel. int*%): *m/z* 426 [M]^+^ C_30_H_50_O (33), 411 [426-15]^+^ (100), 393 [411-18]^+^ (33), 379 [393-15]^+^ (9), 300 (12), 259 (18), 173 (30). ^1^H NMR (500 MHz, CDCl_3_): δ_H_ 5.30 ppm (1H, d, *J* = 3.5 Hz, H-7), 5.10 (1H, dd, *J* = 6.8, 7.2 Hz, H-24), 3.24 (1H, m, H-3), 1.68 (3H, s, H-27),1.61 (3H, s, H-26), 0.97 (3H, s, H-28), 0.97 (3H, s, H-29), 0.86 (3H, s, H-30), 0.84 (3H, brs,H-21), 0.81 (3H, s, H-18), 0.74 (3H, s, H-19).^13^C NMR (125 MHz, CDCl_3_): δ_C_ 37.2 (C-1), 27.7 (C-2), 79.3 (C-3), 38.9 (C-4), 50.6 (C-5), 23.9 (C-6), 117.8 (C-7), 145.9 (C-8), 48.9 (C-9), 35.0 (C-10), 18.2 (C-11), 33.8 (C-12), 43.5 (C-13), 51.3 (C-14), 33.9 (C-15), 28.5 (C-16), 52.2 (C-17), 13.1 (C-18), 22.1 (C-19), 35.8 (C-20), 18.6 (C-21), 35.2 (C-22), 25.4 (C-23), 125.1 (C-24), 130.9 (C-25), 17.7 (C-26), 25.7 (C-27), 27.6 (C-28), 27.3 (C-29), 14.7 (C-30) (8). 

*Cycloartenol (3):* Yellow amorphous powder (20 mg) R_f_ 0.5 (5% AgNO_3_-silica gel TLC, DCM: EtOAc 95:5). IR ν_max_ cm^-1^: 3054(=C-H), 1711, 1594, 1421, 1265(C-O), 738, 705; EIMS (*rel. int*%): *m/z* 426 [M]^+^ C_30_H_50_O (58), 411 [M-15]^+^ (100), 393 [411-15]^+^ (70), 378, 365 (22), 286 (29), 175 (30). ^1^H NMR (500 MHz, CDCl_3_): δ_H_ 5.03 ppm (1H, t, *J* = 7.1, 7.4 Hz, H-24), 3.22 (1H, m, H-3), 1.60 (3H, s, H-27), 1.54 (3H, s, H-26), 0.86 (3H, s, H-18), 0.86 (1H, s, H-29), 0.82 (1H, s, H-28), 0.74 (1H, s, H-30), 0.49 (1H, d, *J* = 4.3, H-19), 0.26 (1H, d, *J* = 4.3, H-19). ^13^C NMR (125 MHz, CDCl_3_): δ_C _30.9 (C-1), 29.4 (C-2), 77.8 (C-3), 39.5 (C-4), 46.1 (C-5), 20.1 (C-6), 27.2 (C-7), 46.9 (C-8), 19.9 (C-9), 24.5 (C-10), 25.0 (C-11), 36.4 (C-12), 44.3 (C-13), 47.8 (C-14), 31.8 (C-15), 25.5 (C-16), 51.3 (C-17), 17.0 (C-18), 28.9 (C-19), 34.9 (C-20), 17.2 (C-21), 35.3 (C-22), 23.9 (C-23), 124.2 (C-24), 129.9 (C-25), 16.6 (C-26), 24.7 (C-27), 18.3 (C-28), 14.3 (C-29), 24.4 (C-30) (8).

*1,2-di-O-α-linolenoyl-sn-glycerol (* ***4*** *):* Colorless gum (7.2 mg) R_f_ 0.6 (silica gel TLC, DCM: acetone, 98:2) IR ν_max_ cm^-1^: 3055, 2987, 2305, 1711, 1421, 1265, 738, 705. EIMS (*rel. int. %):m/z* 612 [M]^+^ C_39_H_64_O_5_ (20), 596 (100), 574 (12), 558 (60), 530 (80), 502 (40). ^1^H NMR (500 MHz, CDCl_3_): δ_H_5.31-5.40 ppm (12H, m, olefinic protons H-9′, H-10′, H-12′, H-13′, H-15′, H-16′), 5.26 (1H, m, H-2), 4.3 (2H, dd, *J* = 4.0, 11.9, H-1b, H-3b), 4.15 (2H, dd, *J* = 6.0, 11.7, H-1a, H-3a), 2.80 (8H, dd, *J* = 5.55, H-11′, H-14′), 2.31 (4H, t, *J* = 7.2, H-2′, H-2″), 2.10 (4H, m, H-8′), 2.03 (4H, m, H-17′), 1.60 (4H, m, H-3′, H-3″), 1.25 (m, envelope methylenes H-4′-H-7′), 0.97 (6H, t, *J* = 7.5, H-18′) ^13^C NMR (125 MHz, CDCl_3_): δ_C_ 61.1 (C-1), 67.8 (C-2), 61.1 (C-3), 171.8, 172.2 (C-1′), 33.0, 33.2 (C-2′), 23.8 (C-3′), 28.7, 28.6, 28.2, 28.1, 28.0 (C-4′, C-5′, C-6′, C-7′), 26.2 (C-8′), 130.9, 129.2, 127.3, 127.2, 126.7, 126.1 (olefinic carbons), C-9′, C-10′, C-12′, C-13′, C-15′,C-16′), 24.6, 24.5 (C-11′, C-14′), 19.5 (C-17′), 13.3 (C-18′) (15).

*1-O-linoleoyl-3-O-palmitoyl-sn-glycerol (5):* Colorless gum (9.1 mg) R_f_ 0.58 (silica gel TLC, DCM: EtOAc 96:4).^1^H NMR (500 MHz, CDCl_3_): δ_H_ 5.25-5.35 ppm (4H, m, olefinic protons), 4.07 (2H, dd, *J*=5.6, 11.5, H-1a, H-3a), 4.11 (2H, dd, *J*=4.2, 11.5, H-1b, H-3b), 4.05 (1H, m, H-2), 2.74 (2H, dd, *J*=6.5, H-11′), 2.28 (4H, t, *J* =7.5, H-2′, H-2″), 2.03 (2H, m, H-17′), 1.98 (2H, m, H-8′), 1.56 (4H, m, H-3′, H-3″), 1.19-1.24 (m, envelope methylenes), 0.91 (3H, t, *J*=6.9, H-18′), 0.81 (3H, t, *J*=6.9, H-16″). ^13^C NMR (125 MHz, CDCl_3_): δ_C_ 64.0 (C-1, C-3), 67.3 (C-2), 172.9, 172.8 (C-1′, C-1″), 33.09, 33.07 (C-2′, C-2″), 23.88, 23.85 (C-3′,C-3″), 28.68, 28.63, 28.58, 28.44, 28.34, 28.23, 28.13, 28.11, 28.07 (envelope methylenes), 129.21, 128.99, 126.72 (olefinic carbons), 26.2 (C-8′), 24.6 (C-11′), 19.5 (C-17′), 13.2 (C-18′), 13.1 (C-16″) (15).

*1-O-*α*-linolenoyl-2-O-palmitoyl-sn-glycerol (6):* Colorless gum (16.2 mg) R_f_ 0.5 (silica gel TLC, DCM: EtOAc 96:4). ^1^H NMR (500 MHz, CDCl_3_): δ_H_ 5.25-5.35 ppm (6H, m, olefinic carbons), 5.0 (1H, m, H-2), 4.23 (2H, dd, *J* = 4.5, 11.9 H-1b, H-3b), 4.18 (2H, dd, *J* =5.7, 11.9, H-1a, H-3a), 2.74 (2H, dd, *J *= 5.0, 6.6, H-11′), 2.70 (4H, dd, *J* = 6.4, 6.7, H-14′), 2.28 (2H, t, 7.5, H-2"), 2.25 (2H, t, 7.5, H-2'), 2.00 (2H, m, H-17′), 1.96 (2H, m, H-8′), 1.55 (4H, m, H-3′, H-3″), 1.20-1.25 (m, envelope methylenes), 0.91 (3H, t, 7.5, H-18′), 0.81 (3H, t, 7.0, H-16″).^13^C NMR (125 MHz, CDCl_3_): δ_C_ 61.0 (C-1), 71.1 (C-2), 60.5 (C-3), 172.8 (C-1'), 172.4 (C-1''), 33.3, 33.1 (C-2′, C-2″), 23.9, 23.8, (C-3′, C-3″), 28.7, 28.6, 28.6, 28.6, 28.6, 28.3, 28.3, 28.2, 28.1, 28.0 (envelope methylenes), 26.2 (C-8′), 127.3, 127.2, 127.2, 127.2, 127.1, 127.0, 126.9, 126.9, 126.8, 126.8 (olefinic carbons), 24.6 (C-11′), 24.5 (C-14′), 19.5 (C-17′), 13.2 (C-18′), 13.1 (C-16″) (15).

*Methyl transestrification*

For confirming the identification of the acyl moieties of the diacylglycerides, they were transformed to their methyl ester derivatives by reaction with BF_3_ in MeOH (17). Briefly 2 mg of the compound was added to 500 μL 20% BF_3_ in MeOH in a sealed test tube and then heated on hot water bath (70 °C) for 1 h. To the above solution, 1 mL water was added and the mixture was extracted three times with 3 mL *n*-hexane. The organic layer were mixed and dried over anhydrous Na_2_SO_4_ and evaporated under nitrogen stream. The residue dissolved in 1 mL hexane and then subjected to GC-MS analyses (18).

*GC-MS analysis*

The GC-MS analytical condition was the same as reported previously (18). The methyl esters of the fatty acids resulting from transesterification of compounds 4-6 were identified by comparison of their retention times (19) and mass spectra recorded on GC-MS with those published in the literature (18,20). The results were in good agreement with those obtained from infra-red (IR), EIMS, 1D and 2D NMR spectral data.

## Results and Discussion

**Figure 1:**
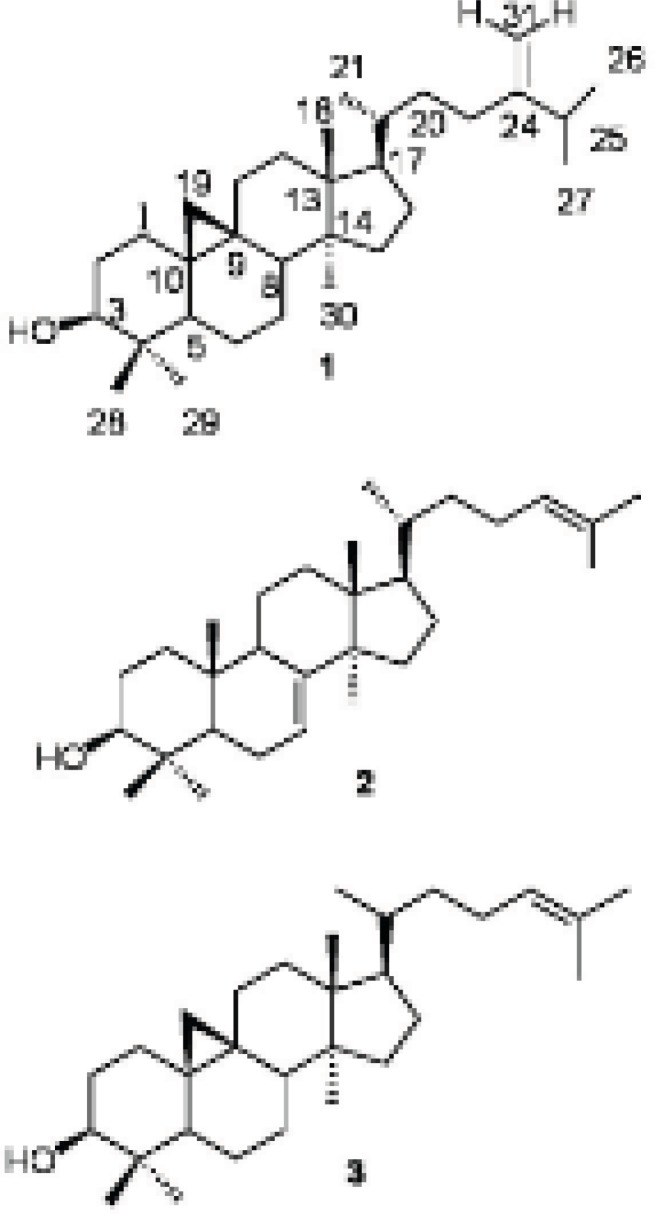
The chemical structure of triterpenoids isolated from *E. macrostegia* (1-3).

**Figure 2:**
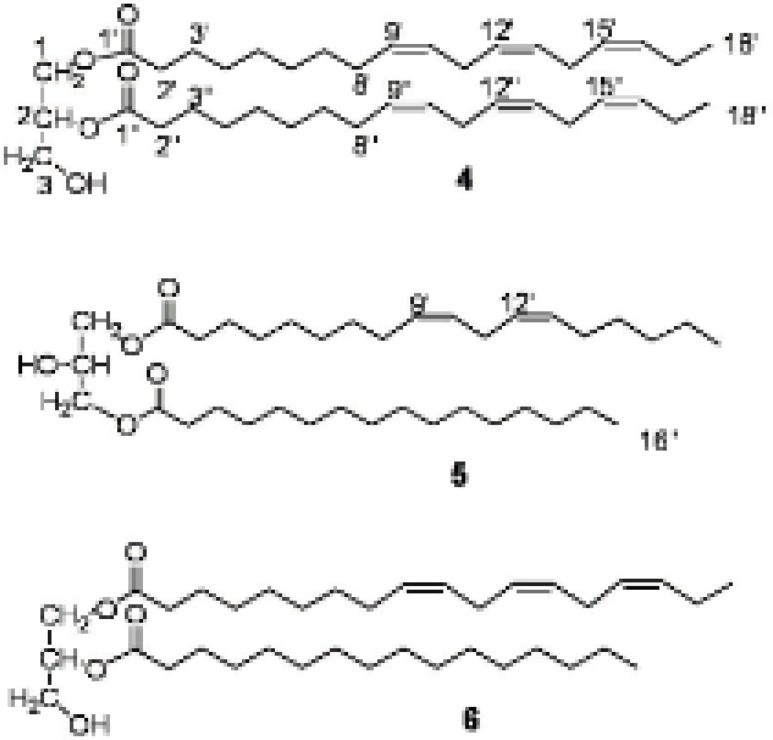
The structure of diglycerides (4-6). The substitution of the esters on C-1 and C-2 of 6 may be interchanged.

From the hexane soluble part of the dichloromethane (DCM)-methanol (MeOH) extract of *E. macrostegia*, three triterpenoids, namely, 24-methylenecycloartan-3β-ol (1), butyrospermol (2) and cycloartenol (3) were isolated using silica gel column chromatography (70-230 mesh) and repeated FCC on AgNO_3_-silica gel (230-400 mesh). In addition to the above mentioned triterpenoids, from the semi-polar fractions of the first column chromatography, three different diglycerides of fatty acids, 1,2-di-*O*-α-linolenoyl-*sn*-glycerol (4), 1-*O*-linoleoyl-3-*O*-palmitoyl-*sn*-glycerol (5) and 1-*O*-α-linolenoyl-2-*O*-palmitoyl-*sn*-glycerol (6) were purified by FCC on silica gel (23-400 mesh). The structure of the compounds were determined by IR, EI-MS and the ^1^H and ^13^C NMR spectral data for compounds 1-3 which were confirmed by comparison to those reported in the literature (Figure 1) (5,8,12). The positions of esterification on the glycerol moiety of the diglycerides (4-6) were determined by ^1^H NMR and HMBC spectral data (Figure 2). 

Diglycerides (4-6) were isolated from the more polar fraction obtained from the silica gel column chromatography. Their structures were deduced mainly from examinations of 1D and 2D NMR spectra. In the ^1^H NMR and ^13^C NMR spectra of different glycerides, the approximate positions of the acyl groups can be deduced from the chemical shifts of the signals of the glycerol protons and carbons (13,14). The priority of the ^13^C NMR spectral data is that the length and degree of unsaturation of the acyl chain does not influence the chemical shift of glycerol carbons chemical shifts but they may alter slightly the chemical shift of the respective protons (13,14). The ^1^H NMR spectral data of the acyl chains of the glycerides (4-6) were assigned with comparison to those published in the literature (13,15,16).

For compound 4, the molecular ion was detected at* m/z* 612 [M]^+^ in EI-MS. APT ^13^C NMR spectrum showed three signals at δ 61.1, 67.8, 61.1 ppm for the glycerol's C-1, C-2 and C-3 carbon atoms, respectively that confirm the 1,2-diglyceride structure (13). In the ^1^H NMR the signals of the protons of the glycerol moiety appeared at δ 4.3 ppm (2H, dd, *J* = 4.0, 11.9, H-1b, H-3b), 4.15 (2H, dd, *J* = 6.0, 11.7, H-1a, H-3a), and the proton signal of H-2 resonated at δ 5.26 ppm which overlapped with the olefinic protons region of the unsaturated acyl moiety and confirmed the substitution of an acyl group at C-2 OH group. The presence of the olefinic protons of the unsaturated acyl chain resonating at δ 5.3-5.4 ppm (m) and six carbon signals at δ 130.9, 129.2, 127.3, 127.2, 126.7, 126.1 ppm suggested three double bonds in the acyl chain. The ^1^H NMR and ^13^C NMR spectral data of the compound suggested the presence of two linolenyl acyl groups in the molecule. 

In the ^13^C NMR spectrum of compound 5**, **two signals for the glycerol carbon atoms appeared at δ 64.0 (C-1, C-3) and 67.3 ppm (C-2) and the respective protons observed at δ 4.07 (2H, dd, *J* = 5.6, 11.5, H-1a, H-3a), 4.11 (2H, dd, *J* = 4.2, 11.5, H-1b, H-3b) and 4.05 ppm (1H, m, H-2) in the ^1^H NMR spectrum suggested the 1,3-diglyceride substitution (13). On the basis of GC-MS analyses of the transesterified products of the compound, one of the acyl chains was suggested to be linoleic acid and the other was identified as palmitic acid methyl ester. The above finding was compatible with the ^1^H NMR spectral data with observing the terminal methyl group of the saturated chain resonated at δ 0.81 ppm while the value was recorded at 0.91 ppm for the linoleyl moiety in addition to four olefinic protons at 5.2-5.3 ppm (m). In addition to the 1D NMR data, the substitutions at C-1 and C-3 was confirmed by observing the cross peaks between H-1,3, H-2', H-2'' and C-1', C-1'' in the HMBC spectrum.

1,2-diglyceride structure was deduced for compound 6 because of the presence of three signals for glycerol carbon atoms at δ 61.0 (C-1), 71.1 (C-2), 60.5 ppm (C-3) in the ^13^C NMR spectrum. The protons attached to glycerol carbons appeared at δ 5.0 (1H, m, H-2), 4.23 (2H, dd, *J* = 4.5, 11.9 H-1b, H-3b), 4.18 ppm (2H, dd, *J* = 5.7, 11.9, H-1a, H-3a) in the ^1^H NMR spectrum (13). The olefinic protons of linolenic chain resonated at δ 5.2-5.3 ppm and the terminal methyl signals appeared at δ 0.91 ppm while this was 0.81 ppm for that of palmityl terminal methyl. The substitution of the esters were confirmed by observing cross peaks between H-1; H-2' and C-1'; H-2 and H-2'' with the C-1'' in the HMBC spectrum, but the substitution of the esters on C-1 and C-2 may be interchanged. 

Compound 1 was isolated in several members of the genus *Euphorbia* and reported to be cytotoxic against p-388 cells and lymphocytic leukemia system at 2.5 μg/mL and more than 20 μg/mL IC_50_s, respectively (4). It is reported as antiproteolytic (21), and causing a dose-dependent decrease in lymphocyte proliferation which is suggested to be due to the presence of the free C-3-OH group in the molecule (5). It shows pain-relieving activity and anti-inflammatory effect (5).

Compound 2 was purified from “shea-butter” which is prepared from the kernels of the fruit of *Butyrospermum parkii,* an African medicinal tree (22). It could be used in formulation of pharmaceutical compositions such as in tablets, parenteral solutions and ointments to cure acne, skin cracks, in sun and skin creams (22,23). The compound is reported to have hormonal and bactericidal activity (22).

Cycloartenol (3) and 24-methylenecycloartan-3β-ol (1) are the two major constituent of *E. broteri* a herbaceous shrub and according to the taxonomic classification suggested by Ponsinet and Ourisson may be in the same clade as* E. macrosteigia* (8,24).

Different biological activities were reported for linolenic acid and its derivatives such as 1,2-Di-*O*-α-linolenoyl-3-*O*-*β*-galactosyl-*sn*-glycerol which was identified as a superoxide generation inhibitor and decrease the O_2_^-^ level in the HL-60 assay system. Reactive oxygen and nitrogen species are toxic molecules against pathogens in the immunological defense system (25). The compound was also reported as a feeding stimulant for *Plagiodera versiclora* a willow beetle living on *Salix integra* (26).

The anticancer, analgesic, and bactericidal activity of the triterpernoids, 24-methylenecycloartan-3β-ol, butyrospermol and cycloartenol together with antifungal (27) and antibacterial activity (28) of unsaturated fatty acids suggest the Persian Spurge, *Euphorbia macrostegia, *is a new source for extraction of the biological active natural products. Although the three diglycerides (4-6) have not shown the above-mentioned biological activities, but their acyl chains such as linolenic and linoleic acid are known to have important biological activities like antimicrobial and antifungal activity especially against important plant pathogens such as *Rhizoctonia solani*, *Phythium ultimum*, *Pyrenophora avenae* and *Crinipellis perniciosa* (27). The above results encourage us to pay more attention to the biological activity of *Euphorbia macrostegia* in our future investigation.
